# World-Wide Immunoscore Task Force: meeting report from the “Melanoma Bridge”, Napoli, November 30th–December 3rd, 2016

**DOI:** 10.1186/s12967-017-1310-9

**Published:** 2017-10-20

**Authors:** Jerome Galon, Alessandro Lugli, Carlo Bifulco, Franck Pages, Giuseppe Masucci, Francesco M. Marincola, Paolo A. Ascierto

**Affiliations:** 1 INSERM (National Institute of Health and Medical Research), Paris, France; 2 Institute of Pathology, University of Bern, Bern, Switzerland; 3 Earle A. Chiles Research Institute, Robert W. Franz Cancer Research Center, Providence Portland Medical Center, Portland, OR USA; 4 Laboratory of Immunology, Hopital Européen Georges Pompidou, Paris, France; 5 Department of Oncology-Pathology, Karolinska Institute, Stockholm, Sweden; 6 AbbVie Medical Corporation, Redwood City, CA USA; 7 Unit of Melanoma, Cancer Immunotherapy and Innovative Therapy, Istituto Nazionale Tumori “Fondazione G. Pascale”, Via Mariano Semmola, 80131 Naples, Italy

**Keywords:** Immunoscore, Lean management system, Cumulative “Suppression Index”

## Abstract

The predictive accuracy of the traditional staging system is based on disease progression as a tumour cell-autonomous process, but it fails to incorporate the effects of the host immune response. A precise analysis of the immune component of the tumour microenvironment by computer-based analysis may be essential to managing patients better, opening the road to an expertise in this new emerging field. The Immunoscore as a new possible approach in the classification of cancer, designated TNM-Immune, studied in colon cancer patients with predictive and prognostic value. This new scoring system is derived from the immune contexture, and is based on the numeration of lymphocyte populations, both in the core of the tumour and in the invasive margin of tumours. The Immunoscore demonstrated to be quantitative, reproducible and robust. The usefulness of Immunoscore in advanced melanoma cancer patients has been as well demonstrated; the correlation of marker expression profile with clinical outcome is ongoing. More recently, the Immunoscore could be a useful prognostic marker in patients with rectal cancer treated by primary surgery. A multivariable Cumulative “Suppression Index” scoring system has been also studied in Oral Squamous Cell Carcinoma patients: it evaluates both the tumor and stromal microcompartments at the invasive margin and summarizes them into the score, providing an accurate stratification, independent of stage, tumour classification. The introduction of Immunoscore requires a redefinition of the Laboratory system according to the LEAN Management process, which has been already implemented in referral research labs. The definition and test of hundreds of biomarkers, in the tumour contexture represents a definitive scientific progression. However, there is still a need of substantial body of work to reach the end of the tunnel to assure a personalize treatment.

## Introduction

The most used system for classifying the extent of cancer is the American Joint Committee on Cancer/Union Internationale Centre le Cancer (AJCC/UICC) Classification of Malignant Tumours (TNM) classification, staging the tumour based on clonal tumour progression as reflected by the degree of locoregional involvement and on the acquisition of a set of unfavourable characteristics, such as the ability to invade lymphatic or blood vessels or to metastasize to distant sites. In daily practice and in guidelines, the TNM category is directly linked to treatment strategies and, as such, changes in the TNM staging system have a considerable and direct impact on the cancer care that patients receive [1].

Unfortunately, the predictive accuracy of the traditional staging system assumes that disease progression is a tumour cell-autonomous process, but it fails to incorporate the effects of the host immune response. On the contrary, it is currently known that tumour progression should now be considered as the result of balance between an invasive tumour process and defence system whose major component is constituted by the host immune response [1]. Galon et al. proposed several ways to classify cancer based on tumour cell characteristics, including morphology, molecular pathways, mutation status, cell origin and gene expression-based methods, giving the possibility to distinguish among multiple, often overlapping, subtypes [1].

Modern classification of tumours is based on the recognition of disease entities that are characterized by morphological, phenotypical, and genetic markers. Each classification system needs to be reliable, reproducible, clinically relevant, and biologically meaningful. Many hurdles have to be taken into consideration, among them: (1) presence of non-neoplastic cells, including immune cells, making the ‘tumour areas’ not ‘pure’ DNA (or RNA) areas and not solely comprised of neoplastic cells; (2) the requirement of immunohistochemical techniques to enable the precise characterization of specific tumour-infiltrating cells; (3) the need to select specific markers [1]. Recent publications indicated that a precise analysis of the immune component of the tumour microenvironment by computer-based analysis may be essential to managing patients better, opening the road to an expertise in this new emerging field.

The Worldwide Immunoscore consortium, including 23 Centres in 17 countries, for more than 3000 patients, composed of international expert pathologists and immunologists, identified a strategy for the organization of worldwide retrospective study for the validation of the Immunoscore in colon cancer. It defined, in different meetings held worldwide in the period December 2012–December 2015, with the support of the World Immunotherapy Council (WIC), of the Society for Immunotherapy of Cancer (SITC) and several other societies, the Immunoscore as a new possible approach in the classification of cancer, designated TNM-I (TNM-Immune) [1].

This new scoring system is derived from the immune contexture, and is based on the numeration of two lymphocyte populations (CD3/CD45RO, CD3/CD8 or CD8/CD45RO), both in the core of the tumour and in the invasive margin of tumours, as a clinically useful prognostic marker in colorectal cancer. The Immunoscore provides a score ranging from Immunoscore 0 (I0) when low densities of both cell types are found in both regions, to Immunoscore 4 when high densities are found in both regions.

This test has a dual advantage: first, it appears to be the strongest prognostic factor for disease free survival (DFS), disease-specific (DSS) and overall survival (OS), including early-stage colorectal cancers; and second, it has biological meaning (adaptive immune response to tumours) and provides a tool or a target for novel therapeutic approaches, including immunotherapy (as recently illustrated in clinical trials boosting T cell responses with anti-CTLA4, anti-PDCD1 (PD-1) and anti-CD274 (PD-L1). Current immunohistochemical technologies allow the application of such analyses in routine diagnostic pathology [1]. The main Immunoscore characteristics are reported in Table 1.

**Table 1:** Characteristics of a good marker and of the Immunoscore [8]

Characteristics	
Routine	Technic to be performed by pathologist using bright field and precise cell evaluation
Feasible	Established pathology technics, using 2 regular whole slide FFPE section
Inexpensive	Automatized immunohistochemistry
Rapid	2 simple staining less costly than complicated molecular techniques
Robust	Autostainers, scanner, and digital pathology reduce the time to perform an Immunoscore
Reproducible	Two strong membrane staining, with no background, allowing the numeration of individual cells
Quantitative	Inter-observers variability is removed by the use of digital pathology, taking into account cell location and counts
Standardized	Standardized operating procedure should be performed to insure reproducibility and worldwide comparisons
Pathology-based	Necessity of pathologist expertise to validate cell type, cell location, and cell counts performed by digital pathology
Powerful	The Immunoscore has a prognostic value highly significant even in Cox multivariate including TNM classification

Unpublished data (Galon et al. 2016) demonstrated the feasibility, reproducibility, significance, and robustness of the Immunoscore in a worldwide study with a statistical analysis pre-defined plan (all statistical analyses performed by external statisticians). The study primary endpoint was time-to-recurrence (TTR) for Immunoscore (High/Low) and inclusion criteria were colon cancer, stages I/II/III (T1–T4, N0–N2, M0), no neo-adjuvant treatments, clinical data and follow-up; exclusion criteria were rectum cancer stages IV (M1), neo-adjuvant treatments, missing clinical data, missing follow-up, staining intensity < 152 and missing/incomplete biomarker data. 3855 patients were quantified for Immunoscore and 2667 patients were analysed after QC and exclusion following a pre-defined statistical analysis workplan. More than 352,000,000 CD3+ T cells were counted by all centres (Table 2).

**Table 2:** Biomarker characteristics results

	Number of CD3+ T cells/slide	Whole slide density of CD3+ (cells/mm^2^)	Whole slide density of CD8+ (cells/mm^2^)
Center	64,537 ± 80,962	685 ± 1297	239 ± 534
Margin	23,643 ± 23,524	1174 ± 1985	436 ± 832
Total	88,180	–	–

Distribution of Immunoscore across all centres: High Immunoscore: 26%; Int. Immunoscore: 49%; Low Immunoscore: 25%

Whole slide quantification demonstrated the best correlation and reproducibility, proving that the Immunoscore is quantitative, reproducible and robust. The primary objective was reached: Immunoscore predicted time to recurrence on training set (TS), and on two independent validation sets (IVS and EVS), blinded to clinical outcome. Besides, multiple secondary objectives are reached Immunoscore 3 groups (and 5 groups); secondary objective as time to recurrence for Immunoscore (High/Int/Low) in Stage II objective was also reached. Immunoscore (2, 3, or 5 groups) is significant in multivariate analyses in TTR; similar results are found for DFS and OS. Immunoscore is significant in multivariate analyses in OS, DFS, TTR (including MSI, T-stage, N-stage, age, gender). In conclusion, the primary endpoint of the Worldwide pre-specified Immunoscore study was reached; the TTR was significantly longer in patient’s stages I/II/III with High-Immunoscore; Low-Immunoscore identified a subgroup of patients with high-risk stage II colon cancer; Immunoscore was significant in multivariate analysis in all cohorts, TS, IVS and EVS and predicted TTR, DFS and OS.

The results of this international consortium may result in the implementation of the Immunoscore as a new component for the classification of cancer, designated TNM-I (TNM-Immune). This will represent the first standardized immune-based assay for the classification of cancer in the era of immunotherapy and will enable the classification cancer patients based on immune parameters.

Immunoscore will impact all types of cancer assessment. For instance, Berghoff et al. assessed the tumor-infiltrating lymphocytes (TILs) in brain metastases (BM), one of the most common and devastating complication of cancer [2] by TIL subsets and their prognostic impact in 116 BM specimens using immunohistochemistry for CD3, CD8, CD45RO, FOXP3, PD1 and PD-L1. Immunoscore predicts overall survival and long-term survival in BM patients (HR 0.612, p < 0.001) [2].

Besides, findings indicate that assessment of the immune status via Immunoscore provides a potent indicator of colorectal tumour recurrence beyond Microsatellite-instability (Min) staging that could be an important guide for immunotherapy strategies. Unpublished data from Mlecnik et al. reported that high immune cell densities and Immunoscore within the Min metastasis predicts prolonged survival.

**Fig. 1:**
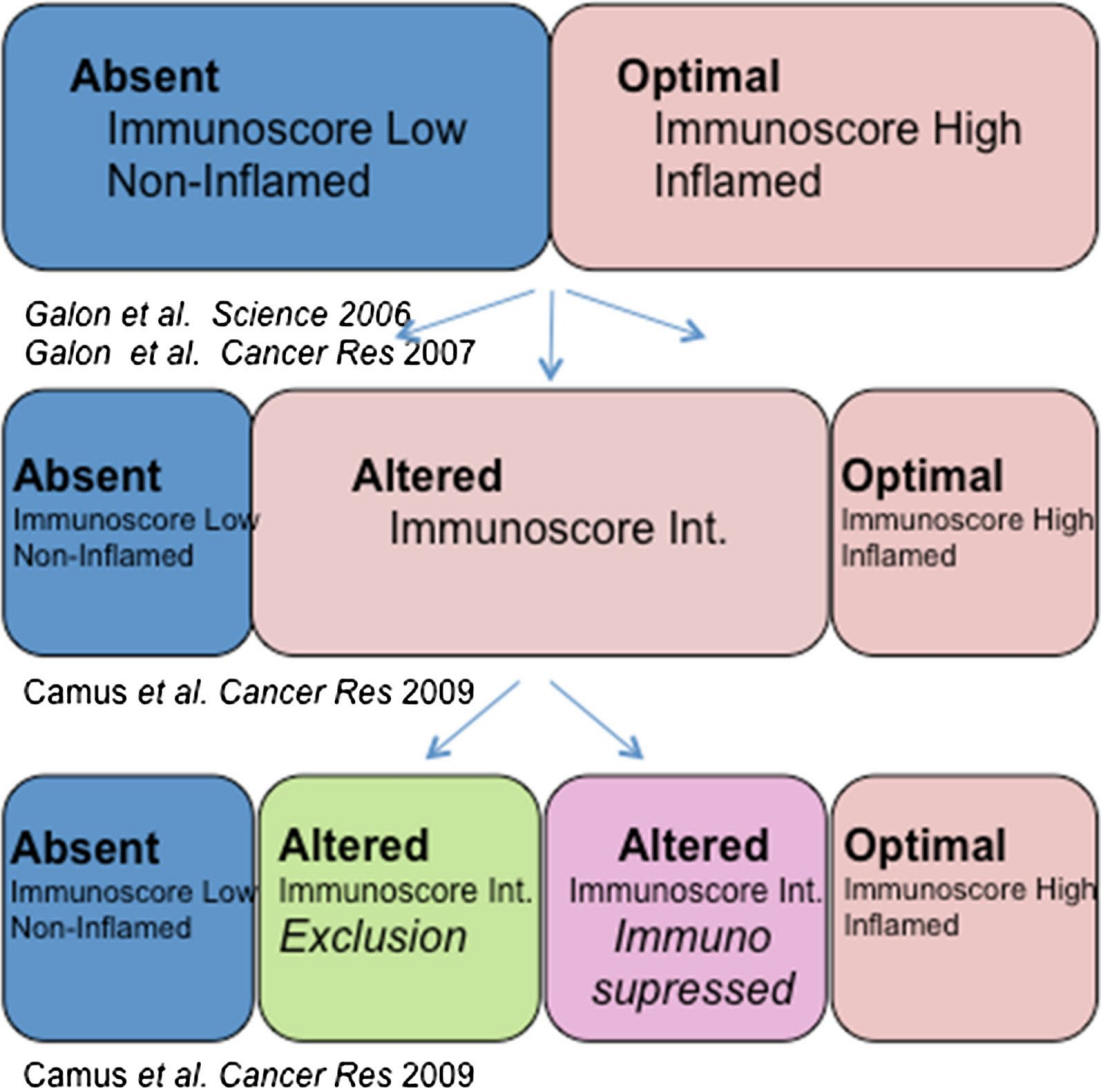
Major immune groups [3–6, 9, 10]

It was demonstrated that the pre-existing immunity and the immune contexture predicts cancer survival [3] and allows to figure out major immune groups of patients (Fig. 1).

The stratification of cancer based on the immune status underlines the importance of having standardized immune assays. Dr. Galon invited to collaborate at the evaluation of the role of Immunoscore on multiple cancers, in particular (among others) Melanoma, Cervical, Uterine, Liver, Pancreas in the Large retrospective cohorts (at all stages) with access to FFPE blocks, available clinical database and 5-year follow-up in order to review articles on Immunoscore in different cancer types, enhancing the topic of immune infiltrates as markers for tumor prognosis and/or response to therapy, stimulate international collaborations between research and clinical investigators worldwide.

### Immunoscore in melanoma

Might Immunoscore and Immunoprofiling be useful in melanoma for selecting patients? The subject has been studied since 1996 [4, 5] and the answer is definitively positive. Dr. Ascierto summarised Immunoscore and Immunoprofiling roles, underlining Immunoscore major utility (Table 3).

**Table 3:** Immunoscore and Immunoprofiling differences

	Immunoscore	Immunoprofiling
	Prognostic/predictive (?)	Prognostic/predictive (?)
**Number of Immunomarkers**	2–4	1-several
**Immunoscore markers**	CD3/CD8	Immune gene signatures
**Immunoscore-like markers**	CD3/CD8/CD20/FoxP3	Multiplex assays
	CD3/CD8/CD45RO	CD137, Galectin1, LAG-3, OX40 PD-L1, TIM3 ect.
	CD3/CD8/CD68	
	CD3/CD8/CD20	
	CD3/GZMB	
	CD8/FoxP3	
	CD8/IL17	
	(others)	
**Possible applications**	• Staging in colorectal cancer (already tested)	• Prognostic assay
	• Staging in melanoma, breast cancer, Ovarian cancer, NSCLC, prostate cancer, pancreatic cancer, head and neck cancer (to be defined)	• Diagnostic assay
		• Personalized immune-treatment

The potential prognostic value of CD3, CD8, CD20, and FOXP3 as an ‘Immunoscore’ for melanoma patients would utilize widely accessible, standardized technology panel and conclude that it could be useful in defining the Immunoscore, because of its prognostic value in high risk melanoma patients. The prognostic and predictive value of the Immunoscore in patients with advanced melanoma was furthermore assessed when treated with Ipilimumab in the previous 2–3 years [6]. The correlation of marker expression profile with clinical outcome is ongoing. A trend for the CD163+ PD-L1+ (CT) population (p = 0.07) is reported but no relationship with response/benefit [6]. A joint project between the National Cancer Institute Pascale in Naples and the manufacturer Definiens assessed the correlation of cell density patterns with Ipilimumab patient overall survival. Prediction of survival based on immune biomarkers before and during treatment reported that before the treatment, short survival patients showed low frequencies of circulating CD16+ CD56dim NK cells, high frequency of TIM-3+ CD56 bright NK cells, high expression levels of TIM-3 on both CD56 dim and CD56bright NK cells. After third cycle of treatment, it showed high TIM-3 expression on CD8+ T cells and on CD56dim NK cells inversely correlated with survival, the increased frequency of KIR+ CD56dim circulating NK cells was associated with adverse prognosis, the increased frequency of CD16+ CD56dim NK cells at this time point strongly correlated with good prognosis and long survival.

### The Immunoscore for the immunomonitoring of colorectal cancer patients and Immunoscore beyond cancer

After years of controversy, immunotherapies have become the hot new thing in cancer drug development and Immunoscore is going to be implemented in the clinical practice, with the great support of clinicians and statisticians too. A prospective multicentre French study (NCT01688232) has been completed: 650 patients with colorectal cancer included, with median follow-up > 3 years (> 100 parameters monitored) to promote the Immunoscore in routine clinical settings.

In a first step, it has been decided not to consider rectal tumours due to distinct clinic pathologic features, tumor markers, and treatment regimens when compared with colonic adenocarcinomas. More recently, Anitei et al. demonstrated that the Immunoscore could be a useful prognostic marker in patients with rectal cancer treated by primary surgery. The determination of the immune infiltrate in biopsies before treatment could be a valuable information for the prediction of response to pCRT [7].

The positioning of Immunoscore in colorectal patients is possible:

1. Patients with Low Immunoscore (high risk of relapse): more intensive treatment (stage II and stage III patients);
2. Patients with High Immunoscore (low risk of relapse): more lighter treatment (e.g. Folfox 3 months versus 6 months);
3. Evaluation of the risk of relapse of stage III patients with contraindication to chemotherapies.

A study is ongoing about the use of Immunoscore to predict non-cancer-related survival time: this is indicative of the host capacity to respond to immune challenges. This capacity could influence the incidence and/or severity of a large range of pathologies. The evidence seems to point towards an association of high immune infiltration means no longer non-cancer-related survival times and patients with a high density of cells for the adaptive immunity implies longer non-cancer-related survival times; finally, patients with a high immune infiltration plus a significant number of lympho nodes, implies a very long-life expectancy. A possible major change of paradigm: the immune system is now recognized not only as a major player in the control of the tumour process but could also be integrated in the clinical practice to predict the outcome and influence treatment decisions beyond the cancer.

The discussion of Dr. Masucci from the Karolinska Institute provided interesting news about Immunoscore in the colorectal cancer. The prognostic and predictive impact of the Immune profile in colon cancer patients stage II and III was assessed in the Stockholm Cohort randomized to surgery or surgery and adjuvant treatment. Genomic amplification of Immunohistochemistry was used to detect HLA-A2, MHC class I, HLA-G, MSI Her-3, CD8+ lymphocytes; time to relapse per age and per gender were assessed and no significant difference was found. Difference between stage II and III was significant and treatment did not change the outcome as far as surgery alone versus surgery and adjuvant treatment.

Min has a relevant prognostic value in women but not in men in terms of time to relapse. Besides, CD8+ score is a stronger independent prognostic factor upon microsatellite instability scoring in women.

As far as HLA-A02 genotype and treatment, stage III patients with HLA-A02 genotype randomized to surgery had a worst outcome; this was not the case for patients with another HLA-A genotype. In the relation among HLA-A02 genotype, gender and treatment, the categories women, stage III, HLA-A02 genotype benefit to adjuvant cytostatic treatment but CD8+ infiltration prevent relapse of the tumour in women only in stage II women. Women treated with adjuvant treatment after surgery presenting high infiltration of CD8+ cells have the highest benefit. HLA-A status is more important than CD8+ for female subgroup.

Finally, CD8+ infiltration is predictive for survival in the all cohort (Table 4).

**Table 4:** CD8+ infiltration and survival (all cohort)

	HR	95% CI	p
HLA-Ax versus HLA-A2	0.859	0.516–1.430	0.5581
CD8+ infiltration at margin and in the tumour HIGH infiltration versus LOW	0.292	0.123–0.695	0.0054

Absence of HLA-A02 genotype is predictive for good survival in the female subgroup compared to CD8+ (Table 5).

**Table 5:** CD8+ infiltration and survival (female)

	HR	95% CI	p
HLA-Ax versus HLA-A2	0.405	0.160–1.027	0.5569
CD8+ infiltration at margin and in the tumour HIGH infiltration versus LOW	1281	0.246–6.679	0.7686

### Implementation of the Immunoscore in a pathology lab based on the lean management system

Lean Management is a management philosophy developed from Toyota Production System focused on improving process speed and quality through reduction of process wastes. Basic concept for Lean management is a business methodology which aims at providing a new way to think about how to organize human activities to deliver more benefits to society and value to individuals while eliminating waste. It is therefore a systematic tool to eliminate waste which is caused by overburden and unevenness of work load.

The application of that to the cancer diagnosis and biomarkers, whose assessment is often characterized by small sample size, incomplete and not reproducible data, should be possible. In fact, an optimal biomarker study should be hypothesis driven, reproducible, with prognostic and/or predictive power and cost-effective.

Some questions raise in the era of personalized healthcare, molecular medicine, digital pathology and automation: where will you implement the Immunoscore? Who is planning? Who is staining? Who is scanning? Who is analyzing? Who is controlling? Who is paying? In order to manage this personalized healthcare revolution a change of culture is required in pathology and it should involve creativity, leadership, and innovation.

The reorganization of the lab of the Institute of Pathology, University of Bern, Bern, Switzerland, according to the Lean management, at implementation, allowed: (1) at operating level, the optimization of the rooms and working places, the optimization of the working process and the continuous working flow; (2) in the diagnostic wing, in the six standardized sign-out rooms, optimal teaching and quality control; (3) in the Lean lab, working with the LEAN Tissue Street (LTS) defined as LEAN Biopsy Street (LBS) and LEAN Resection Street (LRS).

**Fig. 2:**
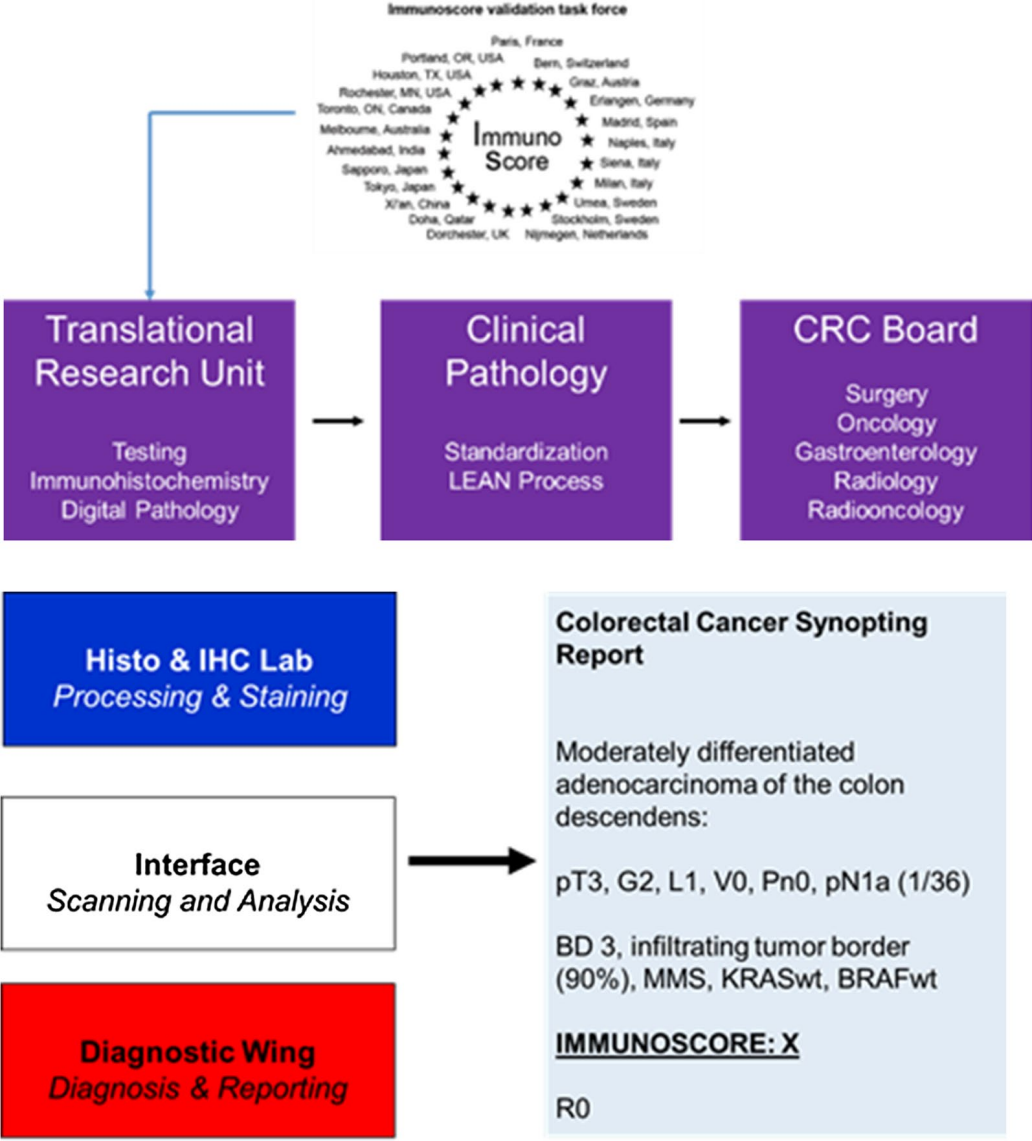
Lean Management applied to the Immunoscore

The Lean management approach allows to implement the Immunoscore workflow, according to the here below reported interactions (Fig. 2).

### Suppressive Index: another side of the Immunoscore

The Immunoscore developed by Galon and colleagues, quantitative assessment of CD3+ and CD8+ T cells at the invasive margin and tumor center is revolutionizing the field of predictive and prognostic biomarker discovery of immune subsets within the tumor, and encouraging the adoption of digital pathology tools for biomarker discover and validation. Currently, CD8+ T cell infiltrates have been shown to have prognostic value in various types of cancer, including melanoma, NSCLC, RCC and bladder cancer. Quantitative IHC assessment can also be predictive, as high densities of CD8 and PD-L1 staining correlate with response to anti-PD-1 immunotherapy agents. However, many patients, with high levels of T cell infiltrates in their tumors, rapidly progress, and other patients, with tumors that have high PD-L1 expression, don’t respond to anti-PD L1 therapy. Therefore, it can be hypothesized that the tumor is characterized by a complex microenvironment that is difficult to describe with single markers such as CD8+ and PD-L1, and that the utilization of multiparametric analysis to study the interactions and spatial relationships between tumour and immune cell types could describe the complex landscape of the tumour microenvironment, helping to more accurately stratify the patients compared to CD8 and/or CD3 alone.

**Fig. 3:**
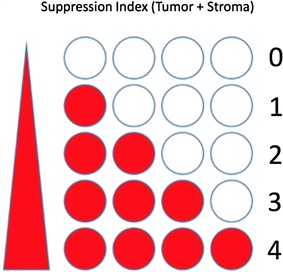
Cumulative Suppressive Index (CSI): multiparametric analysis of the interactions and spatial relationships of both the tumor and stromal microcompartments at the invasive margin

Changes in the composition and function of innate and adaptive immune cells in the tumour microenvironment represent crucial hallmarks for initiation and progression of cancer. In Oral Squamous Cell Carcinoma (OSCC) the composition, frequency and location of peri- and intra-tumoral immune cells, but also the expression of HLA class I antigens and other components of the antigen processing machinery by tumour cells, play an important role in the control of HPV-positive tumours, but the prognostic and therapeutic impact of these parameters in HPV-negative OSCC has not yet been clarified. A multivariable Cumulative “Suppression Index” (CSI) scoring system (Fig. 3) allowed to separate OSCC patients with a 5-year overall survival rate (OSR) of 90 and 20%, respectively. Multispectral imaging enabled the simultaneous evaluation of immune and tumor cells interactions and their use as prognostic biomarkers in evaluating OSCC patients’ outcome, by focusing on the microenvironment centred around CD8+ effector cells. Building on concepts that Galon and colleagues have established previously in their studies on colon carcinoma, the combined “suppression index” evaluates both the tumor and stromal microcompartments at the invasive margin and summarizes them into CSI (Fig. 3). This provided an accurate stratification, independent of stage, separating patients with over 90% 5-year survival from those with only 20% 5-year survival in the study’s cohort. Incorporating tumor levels of beta-2-m, MHC class I HC and LMP10 further improved the prognostic power, identifying 5-year OS rates of 0 to 100% (p < 0.001).

Finally, understanding the nature of T-cell suppressive signals active in the tumor microenvironment may enable a rational approach to combination immunotherapies.

Scientists have been very successful to define and test hundreds of biomarkers, in the tumour contexture, Immunoscore is an example of a validated markers for colon cancer (predictive and prognostic). However, there is still a need of substantial body of work to reach the end of the tunnel to assure a personalize treatment.

## Abbreviations

AJCC/UICC: American Joint Committee on Cancer/Union Internationale Centre le Cancer
TNM: classification of malignant tumours
DFS: disease free survival
DSS: disease-specific
OS: overall survival
TTR: time-to-recurrence
TS: training set
IVS: independent validation sets
TILs: tumor-infiltrating lymphocytes
BM: brain metastases
Min: microsatellite-instability
OSCC: Oral Squamous Cell Carcinoma
CSI: Cumulative “Suppression Index”
